# Epigenetic insights into an epimutant *colorless non-ripening*: from fruit ripening to stress responses

**DOI:** 10.3389/fpls.2024.1440120

**Published:** 2024-07-02

**Authors:** Huihui Zhu, Jian Li Yang, Weiwei Chen

**Affiliations:** ^1^ College of Life and Environmental Sciences, Hangzhou Normal University, Hangzhou, China; ^2^ Key Laboratory of Vegetable Biology, College of Landscape and Horticulture, Yunnan Agricultural University, Kunming, China

**Keywords:** colorless non-ripening (Cnr), crop breeding, epigenetic regulation, tomato, fruit ripening

## Abstract

The epigenetic machinery has received extensive attention due to its involvement in plant growth, development, and adaptation to environmental changes. Recent studies often highlight the epigenetic regulatory network by discussing various epigenetic mutants across various plant species. However, a systemic understanding of essential epigenetic regulatory mechanisms remains limited due to a lack of representative mutants involved in multiple biological processes. *Colorless Non-ripening* (*Cnr*), a spontaneous epimutant isolated from a commercial population, was initially characterized for its role in fruit ripening regulation. *Cnr* fruits exhibit an immature phenotype with yellow skin, attributed to hypermethylation of the *SQUAMOSA PROMOTER BINDING PROTEIN-LIKE-CNR* (*SlSPL-CNR*) promoter, resulting in the repression of gene expression. In addition to DNA methylation, this process also involves histone modification and microRNA, integrating multiple epigenetic regulatory factors. Interestingly, knockout mutants of *SlSPL-CNR* display phenotypical distinctions from *Cnr* in fruit ripening, indicating complex genetic and epigenetic control over the non-ripening phenotype in *Cnr* fruits. Accumulating evidence suggests that *Cnr* epimutation is pleiotropic, participating in various biological processes such as Cd stress, Fe deficiency, vivipary, and cell death. Therefore, the *Cnr* epimutant serve as an excellent model for unveiling how epigenetic mechanisms are involved in diverse biological processes. This review paper focuses on recent research advances regarding the *Cnr* epimutant, delving into its complex genetic and epigenetic regulatory mechanisms, with the aim of enhancing our understanding and facilitating the development of high-quality, high-yield crops through epigenetic modification.

## Introduction

Epigenetic modification refers to alterations in chromatin structure rather than changes in the DNA sequence, encompassing DNA methylation, histones modification, chromatin remodeling and siRNAs (small interfering RNAs). Increasing evidence highlights the vital role of epigenetic regulation in plant growth, development, and stress resilience, as these modifications coordinate with transcription factors to regulate gene expression. For example, the quintuple mutant *mddcc*, lacking DNA methyltransferases such as MET1 (METHYLTRANSFERASE1), DRM1 (DOMAINS REARRANGED METHYLTRANSFERASE1), DRM2, CMT2 (CHROMOMETHYLASE2), and CMT3, displays extreme growth defects including reduced size and failure to transition to the floral stage due to widespread changes in DNA methylation (He et al., 2022). In *Arabidopsis*, mutations in *REF6* (*RELATIVE OF EARLY FLOWERING6*), encoding a histone H3 lysine 27 demethylase, lead to pleiotropic development phenotypes such as early flowering and upward curling leaves upon overexpression (Lu et al., 2011). Additionally, under salinity stress, the DNA methylation reader OsSUVH7 regulates *OsHKT1;5* (*HIGH-AFFINITY POTASSIUM TRANSPORTER1;5*) gene expression by mediating DNA methylation in the upstream transcriptional factors (Wang J. et al., 2020). Clearly, plants rely on epigenetic modifications for normal growth and adaptation to environmental changes.

Epigenetic modifications, such as DNA methylation, histones modification, and siRNAs, are interconnected rather than independent. For instance, the establishment of DNA methylation through the RNA-directed DNA methylation (RdDM) pathway involves siRNAs, which are associated with SUVH2/9 (SUPPRESSOR OF VARIEGATION 3–9 HOMOLOG PROTEIN2/9)-mediated H3K9me2 and the SWITCH/SUCROSE NONFERMENTING (SWI/SNF) chromatin-remodeling complex (Zhang et al., 2018). Thus, DNA methylation emerges as a major epigenetic modification by coordinating with and influencing other epigenetic modifications. However, our current understanding of how DNA methylation regulates plant growth, development, and stress responses primarily stems from studies on various DNA methylation-related mutants across different plant species. Therefore, integrating knowledge of DNA methylation with other genetic and epigenetic components to orchestrate growth and stress responses remains an ongoing challenge.

The epimutant *Cnr* (*Colorless Non-ripening*) in tomato initially demonstrated DNA hypermethylation in the promoter of *SlSPL-CNR* (*SQUAMOSA PROMOTER BINDING PROTEIN-LIKE-CNR*), resulting in the characteristics non-ripening phenotype of fruits (Manning et al., 2006). Subsequent investigations identified that SlCMT3 is responsible for mediating DNA hypermethylation in the *SlSPL-CNR* promoter, along with microRNA SlymiR157 (Chen et al., 2015a, 2015b). Furthermore, the ripening-related transcription factor FRUITFULL1 (FUL1, also known as TDR4), interacting with RIN (RIPENING-INHIBITOR) to regulate fruit ripening (Leseberg et al., 2008), undergoes H3K27me3 modification in the *Cnr* epimutant (Gao et al., 2019). These findings suggest that the *Cnr* epimutant integrates multiple epigenetic regulatory factors, including DNA methylation, microRNAs, and histone modifications, to regulate fruit ripening. Beyond fruit ripening, the *Cnr* epimutant has been implicated in various biological processes, such as Cd stress, Fe deficiency, vivipary, and cell death (Chen et al., 2018, 2022; Lai et al., 2020; Yao et al., 2020). Therefore, summarizing the pleiotropic effects of the *Cnr* epimutant contributes to a comprehensive understanding of epigenetic regulation across different biological processes in plants. This review aims to consolidate recent advancements concerning the *Cnr* epimutant as a representative case study, enhancing our comprehension of the multifaceted roles of epigenetic modifications in diverse biological processes. Ultimately, this understanding may illuminate new avenues for crop breeding by manipulating epigenetic modifications in the future.

## *Colorless non-ripening* epimutant: its past and present

Tomato (*Solanum lycopersicum*) serves as an exceptional model crop for understanding the intricate process of fresh fruit ripening process, involving notable transformations in color, flavor, and texture. Being a climacteric fruit, tomato ripening is marked by a surge in respiration following an increase in ethylene biosynthesis (Biale, 1964; Alexander and Grierson, 2002). Among the naturally occurring mutants in tomato, *Cnr* stands out as an epimutant, initially identified in a commercial population of F_1_ hybrid cv Liberto plants in 1993. The distinct characteristics of *Cnr* fruit include non-ripening with yellow-colored pericarp tissues, along with reduced ethylene production and suppressed softening (Thompson et al., 1999). Furthermore, two additional spontaneous mutants, *ripening inhibitor* (*rin*) and *non-ripening* (*nor*), have been identified, each exhibiting similar rare ripening phenotypes to *Cnr*. These mutations, along with the *Cnr* epimutation, are regarded as major regulators functioning upstream of ethylene-mediated ripening pathways, thereby modulating the expression of numerous ripening-related genes (Vrebalov et al., 2002; Manning et al., 2006; Giovannoni, 2007). It is worth noting that *Cnr* epimutant represents an epigenetic variation, distinct from the gain-of-function genetic variation observed in *rin* and *nor* mutants. Notably, studies have shown that the fruit phenotype in heterozygous lines of hybrid progeny between *Cnr* and *rin* or *nor* does not significantly differ from that of *Cnr* alone, indicating the dominance of the *Cnr* allele among these three spontaneous mutations (Thompson et al., 1999; Wang R. et al., 2020). These findings highlight the pivotal role of *Cnr* epimutation in fruit ripening, positioning it as a master regulator.

Following the genetic investigation, biochemical analysis was conducted to elucidate the implications of *Cnr* epimutation-mediated non-ripening. The pericarp tissue of *Cnr* mutants displayed a depletion in carotenoid pigments and diminished cell-to-cell contacts (Thompson et al., 1999). Further cellular analysis revealed modifications in the middle lamella homogalacturonan within *Cnr* fruits, which result in impaired adhesion to calcium and a thicker cell wall throughout the pericarp compared to wild-type Ailsa Craig (AC) fruits (Orfila et al., 2001). Moreover, ripening-associated soluble pectic polysaccharides in *Cnr* fruits were found to be reduced in comparison with wild-type AC fruits at various ripeness stages (Orfila et al., 2002). These findings reveal a close link between the non-ripening phenotype of *Cnr* fruits with their cell wall properties. Moreover, changes in enzyme activity and gene expression associated with the cell wall were observed during the ripening process of *Cnr* fruits (Eriksson et al., 2004). Specifically, chitinase and peroxidase activity were enhanced in *Cnr* fruits compared to wild-type AC plants. However, ripening-related gene expression was profoundly inhibited in *Cnr* fruits. While these studies elucidated the non-ripening phenotype of *Cnr* fruits from physiological and biochemical aspects, the molecular understanding behind the nature of the *Cnr* epimutant remains lacking.

To delineate the molecular framework underlying the *Cnr*-mediated ripening process, the *Cnr* gene was subsequently characterized. Through positional cloning, the SBP-box (SQUAMOSA promoter binding protein-like) gene was identified at the *Cnr* locus, revealing hypermethylation in the 286-bp promoter region located 2.4 kb upstream from the initiation codon of *SlSPL-CNR* (Manning et al., 2006). This discovery strengthened our comprehension of the key role played by the *SlSPL-CNR* gene and epigenetic variation in tomato fruit ripening. Subsequently, whole-genome bisulfite sequencing was performed on AC and *Cnr* fruit, spanning from the immature stage to the ripe stage, elucidating the dynamic epigenome throughout the fruit ripening process (Zhong et al., 2013). Meanwhile, the promoter region binding transcription factors in fruit ripening regulatory process, such as RIN, were found to undergo demethylation, indicating that DNA methylation, in cooperation with transcription factor binding, is essential for the fruit ripening process. Surprisingly, the CRISP/Cas9-induced knockout mutant of *SlSPL-CNR* failed to replicate the non-ripening phenocopy of *Cnr*, implying that *SlSPL-CNR* contributed little in controlling *Cnr* fruit ripening (Gao et al., 2019). Overall, the traits of the *Cnr* epimutant may be attributed not only to *SlSPL-CNR* itself but also to DNA hypermethylation in other loci, indicating the epigenetic diversity of the *Cnr* epimutant during the fruit ripening process. Therefore, effectively harnessing the diverse epigenetic mechanisms of *Cnr* non-ripening phenotype to facilitate fruit breeding for postharvest quality poses a significant challenge in the future.

## The multifaceted role of *SlSPL-CNR* during fruit development

Initially, the *Cnr* locus was identified within *SlSPL-CNR*, where DNA hypermethylation was observed in its 286-bp promoter region. Notably, virus-induced gene silencing (VIGS) experiments illustrated that this hypermethylation correlated with the suppressed expression of *SlSPL-CNR* from the mature green stage to the ripening stage (Manning et al., 2006). Therefore, *SlSPL-CNR* was considered to contribute to the dominant *Cnr* non-ripening phenotype and was also believed to be necessary for ripening in last decades. SlSPL-CNR, a member of the SQUAMOSA PROMOTER BINDING PROTEIN-LIKE family transcription factors (TFs) first identified in *Antirrhinum majus* (Klein et al., 1996), harbors zinc-finger motifs and a monopartite nuclear localization signal deemed crucial for fruit ripening (Lai et al., 2020). The conservation of the canonical SPL core motif (GTAC) for binding the target gene promoter underscores its functional importance (Birkenbihl et al., 2005). However, *SlSPL-CNR* knockout lines generated by CRISPR/Cas9 technology replicated a less pronounced *Cnr* non-ripening phenotype (Gao et al., 2019), challenging previous assumptions about the function of *Cnr* epimutation. Additionally, proteomic analysis unveiled the complexity of *SlSPL-CNR*’s regulatory mechanism during *Cnr* fruit ripening (Zhou et al., 2022).

The involvement of *SlSPL-CNR* in fruit development extends beyond ripening. It modulates the ripening process by binding to the promoter of *SlTCP18*, a gene named after the three first characterized family members: TEOSINTE BRANCHED (TB) 1 from *Zea mays*, CYCLOIDEA (CYC) from *Antirrhinum majus*, and PROLIFERATING CELL FACTORS (PCFs) from *Oryza sativa*. This interaction affects the expression of genes crucial for fruit maturation (Parapunova et al., 2014). Knockout of *SlSPL-CNR* leads to increased flavonoid content within fruits, achieved by repressing *SlMYB12*, a key regulator of flavonoid biosynthesis (Zhou et al., 2023). Moreover, it directly controls genes associated with wax biosynthesis, notably *SlCER1–2* (*ECERIFERUM1*) and *SlCER6* (*β-KETOACYL-COENZYME A SYNTHASE6*), with the aim of reducing postharvest water loss and enhancing fruit quality (Chen et al., 2024). *SlSPL-CNR*’s upstream regulatory network involves intricate interactions with other genes like *RIN* and *SlBL4* (*BELL-LIKE HOMEODOMAIN4*), influencing fruit ripening and chloroplast development (Zhou et al., 2012; Yan et al., 2020). Besides, homolog genes in *Arabidopsis*, such as *AtSPL3*, play pivotal roles in the floral transition (Cardon et al., 2002), expanding the understanding of *SlSPL-CNR*’s functionality. Moreover, recent research reveals that *SlSPL-CNR* impacts cellular homeostasis and stress responses during fruit development by interacting with SlSnRK1 (SUCROSE NON-FERMENTING 1-RELATED PROTEIN KINASE1), thereby implicating it in the regulation of cell death processes (Lai et al., 2020). These findings collectively emphasize the multifaceted regulatory roles of *SlSPL-CNR* in tomato fruit biology.

## The upstream regulatory network in *colorless non-ripening* fruit ripening process

The regulatory mechanism underlying the *Cnr* non-ripening phenotype represent a fascinating intersection of genetic and epigenetic control. Especially, epigenetic modifications, including microRNAs (miRNAs) and DNA methylation, act as upstream regulatory factors and play a significant role in *Cnr* fruit ripening process. MiRNAs, known for their regulatory role in multiple biological processes, including growth, development, and stress responses, emerge as key players in the intricate network governing fruit ripening. Research has highlighted the involvement of miRNAs in controlling the expression of ripening transcription factors through mRNA cleavage during fruit ripening (Moxon et al., 2008; Mohorianu et al., 2011; Karlova et al., 2013). Specifically, SlymiR157 has been identified as directly targeting *SlSPL-CNR*, thereby influencing its expression by inducing mRNA degradation and translational repression, ultimately impacting the ripening process in *Cnr* fruits (Chen et al., 2015a).

In addition to miRNAs, epigenetic modifications, particularly DNA methylation, also play crucial roles in fruit ripening. Screening of genes encoding DNA methyltransferases has revealed the significance of SlCMT3 in the *Cnr* epimutant phenotype. Silencing of *SlCMT3* in this context alleviates DNA hypermethylation observed at the *SlSPL-CNR* promoter and RIN binding sites, resulting in increased *SlSPL-CNR* expression and subsequent restoration of normal ripening in *Cnr* fruits (Chen et al., 2015b). Interestingly, the maintenance of DNA methylation dynamics in the *Cnr* epimutant primarily involves methylated cytosines in CG and CHG contexts. While the role of SlCMT3 in maintaining DNA methylation balance is evident (Chen et al., 2015b), further exploration suggests that SlMET1 might also be critical for sustaining DNA methylation alongside SlCMT3 in the *Cnr* epimutation (Yao et al., 2020). This aspect necessitates comprehensive investigation to elucidate the full scope of the epigenetic regulatory mechanisms governing the *Cnr* non-ripening phenotype.

## *Colorless non-ripening* epimutation is involved in multiple biological process

*Cnr* epimutation participates in various biological processes, including phytohormones production and signaling, cell wall property modification, and extensive modification of the DNA methylome (Thompson et al., 1999; Orfila et al., 2001, 2002; Manning et al., 2006; Zhong et al., 2013). Such diverse involvements suggest a broader role for *Cnr* epimutation in various biological processes and stress responses. In the *Cnr* epimutant, DNA hypermethylation is observed in genes related to abscisic acid biosynthesis, such as *SlNCED* (*9-CIS-EPOXYCAROTENOID-DIOXYGENASE*), resulting in reduced expression of *SlNCED* and consequently diminished abscisic acid biosynthesis in seeds, which coincides with the vivipary phenotype observed in *Cnr* fruits (Yao et al., 2020). In addition, studies have revealed the role of *Cnr* epimutation under abiotic stress conditions. Under Cd stress, the *Cnr* epimutant exhibits increased sensitivity to Cd due to the repression of *SlSPL-CNR* expression, which directly impacts the *SlNR* (*NITRATE REDUCTASE*) promoter, influencing NO production and resulting in the accumulation of more Cd compared to wild-type AC plants (Chen et al., 2018). Considering the essential role of NO in Fe deficiency responses, the *Cnr* epimutant has been implicated in constitutive Fe deficiency responses even under Fe-sufficient conditions, as revealed through comparative physiological and transcriptomic analyses (Chen et al., 2022). Although initial evidence pointed to the importance of *SlSPL-CNR* in Fe-deficient responses in *Cnr* roots, subsequent studies have provided a comprehensive understanding of the epigenetic mechanisms regulating Fe homeostasis by *Cnr* epimutation. Hypermethylation of the *SlSPL-CNR* promoter in *Cnr* roots inhibits its expression (Chen et al., 2022), while SlSPL-CNR protein directly binds to *SlbHLH101* (*BASIC HELIX-LOOP-HELIX101*) promoter, inhibiting its transcriptional expression (Zhu et al., 2022). Additionally, SlymiR157 targets *SlSPL-CNR* expression for mRNA degradation, consequently releasing *SlbHLH101* expression to facilitate Fe-deficient responses (Zhu et al., 2022). Furthermore, SlMET1-dependent CG hypermethylation of the *SlPME53* (*PECTIN METHYLESTERASE53*) intron induces its expression, regulating apoplastic Fe reutilization in the *Cnr* epimutant (Zhu et al., 2024). In summary, the retention of Fe in the apoplast and its uptake into the cytoplasm contribute to the constitutive Fe-deficient response of *Cnr* under Fe-sufficient conditions. Upon perceiving the Fe deficiency signals, retained Fe in the pectin enters the cell for reutilization. By dissecting both genetic and epigenetic factors, these findings systematically unveil the *Cnr*-mediated regulatory mechanisms in response to Fe deficiency in tomato, underscoring the importance of harmonizing the pleiotropy of *Cnr* epimutation for systematic functional exploration in *Cnr* research.

## Prospectives

**Figure 1:**
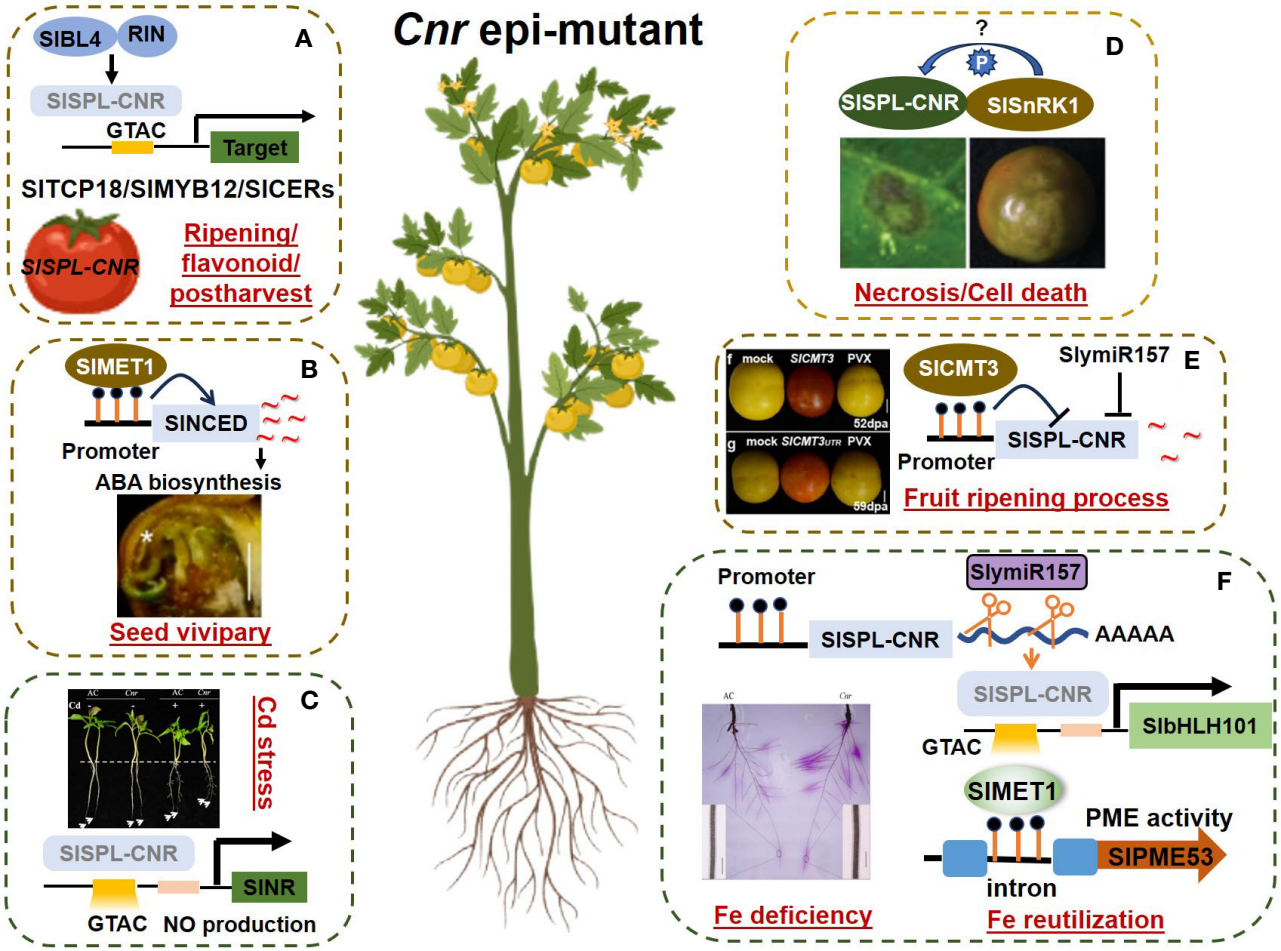
The pleiotropic role of *Cnr* epimutation in various processes. The regulatory network of *Cnr* epimutation in tomato is intricate and multifaceted, contributing to various crucial processes. Here’s an overview of each module and its associated references. Different modules (separated by dashed box) represent different processes. **(A)** The regulatory network of *SlSPL-CNR* in fruits involves ripening, flavonoid synthesis, and postharvest process. This module is supported by studies derived from Parapunova et al.(2014), Zhou et al. (2023) and Chen et al. (2024). SlBL4: BELL-LIKE HOMEODOMAIN4; SlSPL-CNR: SQUAMOSA PROMOTER BINDING PROTEIN-LIKE-CNR; RIN: RIPENING-INHIBITOR; SlTCP18: TEOSINTE BRANCHED & CYCLOIDEA & PROLIFERATING CELL FACTORS18; SlMYB12: R2R3-MYB TRANSCRIPTION FACTOR12; SlCERs: SlCER1–2 (ECERIFERUM1) and SlCER6 (β-KETOACYL-COENZYME A SYNTHASE6). **(B)** *Cnr* mediates vivipary tthrough METHYLTRANSFERASE1, as domonstrated by Yao et al. (2020). SlNCED: 9-CIS-EPOXYCAROTENOID-DIOXYGENASE. **(C)** *SlSPL-CNR* modulates Cd acquisition by targeting and repressing *SlNR* expression, thereby regulating NO production, as reported by Chen et al. (2018). SlNR: NITRATE REDUCTASE. **(D)** The interaction between SlSPL-CNR and SlSnRK1 mediates ripening and cell death, elucidated by Lai et al. (2020). SlSnPK1: SUCROSE NON-FERMENTING 1-RELATED PROTEIN KINASE1. **(E)** Upstream regulators of *Cnr* fruit ripening process, including Manning et al. (2006), Zhong et al. (2013), and Chen et al. (2015a, 2015b), provide insights into the regulatory cascade governing fruit ripening. SlCMT3: CHROMOMETHYLASE3; SlymiR157: microRNA miR157. **(F)** The SlymiR157−SlSPL-CNR−SlbHLH101 model and SlMET1−SlPME53 model are involved in Fe uptake and Fe reutilization, respectively, as described by Chen et al. (2022), and Zhu et al. (2022, 2024). SlMET1: METHYLTRANSFERASE1; SlbHLH101: BASIC HELIX-LOOP-HELIX101; SlPME53: PECTIN METHYLESTERASE53.

The pleiotropic effects of *Cnr* epimutation on various developmental processes and stress responses highlight extensive DNA methylome modification within the *Cnr* genome, giving rise to distinct regulatory mechanisms (**Figure 1**). Bisulfite sequencing has revealed that nearly half of the cytosine positions in the *Cnr* fruit genome are methylated (Zhong et al., 2013). This finding suggests the contribution of not only *SlSPL-CNR* but also other potential epigenetic loci to the non-ripening phenotype of *Cnr*. Therefore, comprehensive big data analyses are warranted to elucidate the genome-wide effects of *Cnr* epimutation in future studies.

Furthermore, the methylome pattern of *Cnr* appears to vary depending on organ type or developmental stages, as evidenced by disparities between *Cnr* fruits and roots in bisulfite sequencing data (Zhu et al., 2024). Considering these observations alongside the diverse effects of *Cnr* on fruit ripening, leaf cell death, and root responses to abiotic stress, it is imperative to account for tissue-specific effects when evaluating the pleiotropic effects of *Cnr*. Moreover, the involvement of MicroRNA SlymiR157 in *Cnr* fruit ripening and the association of the ripening-related transcription factor TDR4/FUL1 with hyperH3K27me3 marks in the *Cnr* epimutant underscore the importance of exploring cooperative mechanisms beyond DNA methylation alone in the *Cnr*-mediated regulatory network across various biological processes. Future research efforts should delve deeper into the crosstalk between genetic regulation and epigenetic regulation, including DNA methylation modification, histone modification, and microRNAs, to enhance our understanding of the intricate regulatory network governed by *Cnr* epimutation.

Lastly, the potential application of *Cnr* epimutant, as representative epigenetic variations, in the development of novel strategies and technologies for crop improvement warrants consideration. For example, an ideal crop could be constructed in concordance with breeding stress-tolerant crop and fruit breeding for postharvest quality after deciphering the epigenetic codes of *Cnr* in stress responses and fruit ripening process, respectively. In summary, a systematic approach integrating multi-omics, tissue-specific, and the exploration of multiple genetic and epigenetic modifications is essential for constructing *Cnr*-mediated regulatory network, ultimately facilitating advancements in improving tomatoes.

## Author contributions

HZ: Conceptualization, Writing – original draft, Writing – review & editing. JY: Supervision, Writing – original draft, Writing – review & editing, Funding acquisition. WC: Funding acquisition, Resources, Writing – original draft, Writing – review & editing.
